# Hemophilia Care in the Pediatric Age

**DOI:** 10.3390/jcm6050054

**Published:** 2017-05-19

**Authors:** Marta Bertamino, Francesca Riccardi, Laura Banov, Johanna Svahn, Angelo Claudio Molinari

**Affiliations:** 1 Thrombosis and Hemostasis Unit, Regional Reference Center for Hemorrhagic Diseases, Giannina Gaslini Children’s Hospital, 16147 Genova, Italy; martabertamino@gaslini.org (M.B.); laurabanov@gaslini.org (L.B.); aclaudiomolinari@gaslini.org (A.C.M.); 2 Hematology Unit, Giannina Gaslini Children’s Hospital, 16147 Genova, Italy; francescariccardi@gaslini.org

**Keywords:** hemophilia, neonate, child, treatment, prophylaxis, vaccinations, sport, psychology, venous access

## Abstract

Hemophilia is the most common of the severe bleeding disorders and if not properly managed since early infancy can lead to chronic disease and lifelong disabilities. However, it enjoys the most efficacious and safe treatment among the most prevalent monogenic disorders. Hemophilia should be considered in the neonatal period in the case of unusual bleeding or in the case of positive family history. Later, hemophilia should be suspected mainly in males because of abnormal bruising/bleeding or unusual bleeding following invasive procedures—for example, tonsillectomy or circumcision. Prophylactic treatment that is started early with clotting-factor concentrates has been shown to prevent hemophilic arthropathy and is, therefore, the gold standard of care for hemophilia A and B in most countries with adequate resources. Central venous access catheters and arterovenous fistulas play an important role in the management of hemophilia children requiring repeated and/or urgent administration of coagulation factor concentrates. During childhood and adolescence, personalized treatment strategies that suit the patient and his lifestyle are essential to ensure optimal outcomes. Physical activity is important and can contribute to better coordination, endurance, flexibility and strength. The present article focuses also on questions frequently posed to pediatric hematologists like vaccinations, day-care/school access and dental care.

## 1. Background

Hemophilia was thought to be a uniform entity for several centuries. The discovery that the coagulation defect in the blood of one hemophiliac could be normalized by infusion of blood from another hemophiliac in 1940 [1] led to finding that there were two types of hemophilia: hemophilia A, due to deficiency of coagulation factor VIII (FVIII) and hemophilia B, caused by the reduction of coagulation factor IX (FIX) [2]. Based on the residual activity of the defective factor, hemophilias were divided into three different degrees of severity: severe, moderate, and mild. In severe hemophilia, the plasma content of coagulation factor activity (FVIII:C for hemophilia A and FIX:C for hemophilia B) is less than 1% of normal, compared with 1 to 4% in moderate hemophilia and 5 to 40% in mild cases [3,4]. Within a given family, the affected subjects always manifest the same type of hemophilia and the same degree of severity [4,5].

Hemophilia A and B are sex linked diseases. The genes responsible for the synthesis of factor VIII and factor IX are located on the X chromosome. The hemophilia trait in women is recessive because a normal X chromosome is also present and responsible for at least a 50% level of coagulation factor VIII or factor IX. If a hemophiliac man with one abnormal X chromosome and a healthy woman with two normal X chromosomes have children, all of their daughters will be hemophilia carriers and all of their sons will be healthy. On the other hand, if a woman who is a hemophilia carrier has children together with a healthy man, a male child is at 50% risk of being affected and a female child is at 50% risk of being a carrier of hemophilia. In approximately one third of the so-called sporadic cases, in which there is no family history of hemophilia, a new mutation on the X chromosome of the factor VIII or IX gene will be found. The prevalence of hemophilia in most countries has been reported to be 13 to 18 per 100,000 men and the ratio between hemophilia A and hemophilia B is about 4:1 [6]. 

Hemophilia is the most common of the severe bleeding disorders and enjoys the most efficacious and safe treatment among the most prevalent monogenic disorders; however, if not properly managed since early infancy, it can lead to chronic disease and lifelong disabilities [6].

## 2. Perinatal Care, Diagnostic Issues and Workup of a Bleeding Child

The severe forms of factor deficiencies, hemophilia A and B, are diagnosed in the neonatal period in 52% and 68% of cases [7,8,9]. In this period, acquired bleeding disorders are more frequent rather than congenital forms of coagulation factor deficiencies. However, there should be a high index of suspicion of congenital disorders in an otherwise healthy neonate who presents with unusual bleeding, since it is estimated from molecular studies that at least 30% of newly diagnosed cases of hemophilia occur as a consequence of a new mutation and thus without a positive family history [10]. The cranium is the most common site of bleeding in neonates with hemophilia, with intracranial hemorrhages (ICH) being 27% of all bleeds and extracranial hemorrhages (ECH) 13%. This is related to birth trauma regardless of delivery mode. Other iatrogenic causes of bleeding included vascular, capillary and intramuscular puncture, and circumcision. Thus, in a neonate with a suspected severe congenital bleeding disorder, such invasive procedures should be avoided until diagnosis can be confirmed or ruled out [11,12,13]. ICH incidence in neonates with hemophilia is reported as between 1% and 4% with possible devastating consequences. Even ECH such as subgaleal hemorrhages can be life threatening due to hypovolemic shock. Early diagnosis of ICH, ECH or other severe bleeding in the neonatal period and prompt coagulation investigation can lead to appropriate therapy and prevention of neurological consequences. In the case of a neonate with positive family history of hemophilia, a cranial ultrasound scan after birth can rule out the presence of ICH and is a non-invasive simple method to screen for pauci- or asymptomatic cranial bleed in these patients [12,14]. Although in a known hemophiliac newborn it is not recommended to administer the missing coagulation factor in order to prevent bleeding after delivery, it should be considered in those with traumatic delivery [11,15].

Diagnosis of hemophilia should be investigated for in the neonatal period as well as outside this period, in case of unusual bleeding or in case of family history of hemophilia. Outside the newborn period hemophilia should be suspected because of abnormal bruising/bleeding or unusual bleeding following invasive procedures—for example, tonsillectomy or circumcision. Other than the coagulation screening, it is fundamental to assay the FVIII and FIX level since the screening tests are physiologically prolonged in the neonatal period [6]. 

## 3. Vaccinations

A child with hemophilia can and should be vaccinated like other children. While most vaccines can be given subcutaneously, some are necessary to give intramuscularly. The World Federation Hemophilia (WFH) guidelines [6] suggest that, if intramuscular injection is necessary, due to some vaccines having only intramuscular route as suggested route of administration, it is best done soon after a dose of factor replacement therapy, an ice pack should be placed over the injection site 5 min before injection, a small gauge needle (23 gauge) should be used and pressure should be applied on an injection site for 5 min after injection. As a common trend and as a hypothetical measure to avoid an immune system challenge and inhibitor formation, a vaccination should not be given on the same day as factor concentrate infusion. On the other hand, the possible role of vaccinations as “danger signal” for inhibitor development has been described [16,17]. However, recent data from the PedNet registry presented by M. Van den Berg, during the annual meeting of the European Association for Haemophilia and Allied Disorders (EAHAD) 2017 [18], showed that vaccination does not pose any increased risk for inhibitor development.

## 4. Product Choice

In developed countries, most children with hemophilia are treated with recombinant FVIII/FIX, mainly due to their perceived higher safety [19,20]. Recently, a randomized study demonstrated that the combined risk of developing a high- or low-titer inhibitor within the first 50 exposure days of using some recombinant factors is 1.87-fold higher than when using plasma-derived factor concentrates [21]. However, some concerns have been raised about the possible implications of these results onto the clinical practice [20,22].

Moreover, very recently, the Pharmacovigilance Risk Assessment Committee (PRAC) of the European Medicines Agency (EMA) concluded that there is no clear and consistent evidence of a difference in the incidence of inhibitor development between the two classes of factor VIII medicines: those derived from plasma and those made by recombinant DNA technology (http://www.ema.europa.eu/docs/en_GB/document_library/Referrals_document/Factor_VIII_31/Recommendation_provided_by_Pharmacovigilance_Risk_Assessment_Committee/WC500226901.pdf).

Newer Extended Half-life Recombinant Agents, also known as enhanced half-life (EHL) clotting factors, may help in the future to reduce the frequency of prophylaxis administration. Common strategies for enhancing the half-life of a molecule include linkage to the fragment crystallizable (Fc) region of Ig-antibody, to polyethylene glycol (PEG) or to recombinant albumin [23,24,25,26,27,28,29].

## 5. Regimen Choice and When to Start

The goal of treatment of a child with hemophilia is to enable the patient and their families to manage the illness as independently as possible and therefore lead more normal, healthy lives. The most important aspect of the care of a child with hemophilia is the treatment regimen that is used.

Prophylaxis with clotting-factor concentrates, initially introduced in Sweden in the 1960s [30] is nowadays the standard of care for hemophilia A and B in anticipation of bleeding or to prevent it. Episodic treatment (“on demand”) is associated with worst outcome [6]. Safe prophylaxis was made possible in 1992 with the approval of the first recombinant factor for replacement therapy, as well as with the improved safety of plasma-derived products [31].

Manco-Jonson et al. in a large cohort prospective study demonstrated the efficacy of prophylaxis with recombinant factor VIII in reducing the incidence of joint hemorrhages, life-threatening hemorrhages, and other hemorrhages and in lowering the risk of joint damage among young boys with severe factor VIII deficiency [32]. However, the high cost of recombinant factor VIII is a barrier to widespread acceptance of prophylaxis [33]. 

Early initiation of primary prophylaxis is ideal, but secondary prophylaxis in adolescents and adults has also demonstrated significant success. Studies have shown that the earlier prophylaxis is initiated, the greater the likelihood of preventing joint damage [23,24,25,26,32].

However, observations have shown significant interindividual variations in timing, dose and frequency requirements of people with hemophilia on prophylactic factor replacement therapy, linked to underlying genetic issues [23] and environmental issues such as lifestyle, venous access, compliance and ability to home and self infusion for example [34]. Personalization of the prophylaxis should be regarded as a dynamic process that requires continuous monitoring and modification according to the actual needs of the individual patient [35]. As an aid in tailoring prophylaxis, a possibility is to use pharmacokinetics as a guide [36]. Given the fact that even a single bleed may initiate joint damage, the ultimate goal of an ideal prophylactic factor replacement treatment should be zero bleeding as suggested by the WFH [37].

To date, the most refined regimen involves primary prophylaxis started before the onset of joint bleeding or other serious bleeds, at 12–18 months of age [6] (Table 1) or earlier as in other centres who start before one year of age [38]. Although the treatment should be individualized, taking into account the patient’s age, venous access, bleeding phenotype, susceptibility to joint arthropathy, and availability of clotting factors, the protocol more commonly used for prophylaxis is as follows: in most cases, an early therapeutic approach is initiated by giving approximately 30–50 IU/kg once or twice a week, with the aim of increasing the frequency of administration as soon as possible, until reaching full-scale primary prophylaxis. In hemophilia A, FVIII is administered at dosage of 20–40 IU/kg/day every second day or three times weekly; in hemophilia B, FIX is administered at dosage of 20–40 UI/kg/day every third day or two times weekly [6,39].

## 6. Inhibitors

The development of inhibitors, which occur in 30–35% of previously untreated children with severe hemophilia A and around 2–5% of patients with severe hemophilia B infused with the available commercial product, remains the major complication of therapy in hemophilia [40,41]. Inhibitors are alloantibodies directed against replacement FVIII or FIX that typically neutralize the activity of the factor [42]. Inhibitors are measured and quantified by the Nijmegen modified Bethesda assay, where one Bethesda Unit (BU) is defined as the amount of inhibitor that will neutralize 50% of one unit of FVIII:C in normal plasma after 120 min incubation at 37 °C [43]. 

The development of inhibitors is one of the most severe complications of hemophilia treatment and it remains an important clinical challenge, with limited treatment options [41]. Although a significant difference in bleeding frequency between patients with or without inhibitors has so far not been demonstrated, the management of bleeding episodes in the presence of high-titre inhibitors is more problematic. Therefore, patients with inhibitor are at particular risk of developing long-term disabling joint damage, uncontrollable bleeding, premature death [44,45] and bleeding episodes negatively affect the Quality of Life (QoL) of patients and their caregivers [46].

Inhibitors tend to develop within the first 20 exposure days (ED, defined as a 24-hour period during which one or more exposures to FVIII therapy was recorded, [6,42]) and rarely develop after 50 exposure days [6,40]. Disease severity, major FVIII gene defects, family history, and non-Caucasian race are major genetic risk factors for inhibitor development [17,47,48]. Low titre inhibitors are defined as <5 Bethesda units (BU)/mL and high titre ≥5 BU/mL [42].

Patients with high-titre inhibitors require individualised therapy with bypassing agents [6], namely, recombinant activated FVII (rFVIIa, NovoSeven^®^, NovoNordis, Bagsvaerd, Denmark) or plasma-derived activated prothrombin complex concentrate (pd-aPCC, FVIII inhibitor bypass activity, FEIBA^®^, Baxalta Shire, Lexington, MA, USA). It was demonstrated that aPCC and rFVIIa are equally effective for the treatment of acute bleeds [49]. Some evidence points out that bypassing agents can also prevent significant percentage of bleedings and would thus justify a prophylaxis regimen with bypassing agent in selected severe cases of high-titre inhibitors [49,50,51,52]. Recently, some new strategies have been demonstrated to be capable of inducing hemostasis in patients with FVIII inhibitors: FVIII mimetic therapy [53], rebalancing hemostasis by anti-thrombin inhibition [54] and inhibition of tissue factor pathway inhibitor [55].

The main goal in patients with inhibitors is to eradicate the antibody; thus, immune tolerance induction (ITI) with high daily doses of FVIII has become the standard therapy; however, no standard dosing schedule has been defined although some data from the i-ITI study and registries favor very high versus very low dosing schedules while additional treatments like immunosuppression are reported only in single cases or small series [56,57,58].

Early detection of an inhibitor is crucial to minimize anamnesis and, if the inhibitor does not rise above 10 BU/mL, allow ITI to be started without delay. In the case of a high-titer inhibitor >10 BU/mL, it is advisable to assume a “wait and see” strategy initially, monitoring the inhibitor titer at least monthly and ideally start ITI once the titer <10 BU/mL or earlier in case of clinical need, bleeding phenotype for example. At our center, we prefer not to wait more than few months to start ITI to avoid severe bleeding complications.

Early detection will also limit exposure to sub-optimal treatment. Inhibitor testing is required before elective invasive procedures, when the clinical or laboratory response to concentrate is sub-optimal, before and after a switch of concentrate and 2–3 weeks after intensive treatment (five EDs) or surgery in mild or moderately affected patients and when the bleeding phenotype of the patient seems worsening [59].

## 7. Venous Access

One of the major issues and difficulties in the treatment of hemophilia in children is the venous access. Easy venous access is a prerequisite for treatment of hemophilic patients with factor concentrates, whether this is prophylaxis, on-demand or an ITI regimen. In the case of on-demand treatment, the child needs to receive the factor concentrate as soon as the bleed occurs, at home by the parents. This implies the necessity of a safe and easy access to a vein. The same is true for children on a prophylactic regimen and even more so for those who are on ITI requiring factor concentrate infusion at least once a day. The alternative to a peripheral vein in the case of on demand or prophylactic regimen, and a necessity in case of ITI, is a central venous access device (CVAD) or an arteriovenous fistula (AVF) [60]. 

The choice of venous access in children with hemophilia depends on the child’s age, the parents’ and caregivers’ choice as well as the familiarity with the devices at the particular hemophilia center. A totally implantable CVAD such as a Port-a-cath carries a reduced infectious risk compared to a peripherally inserted external non-tunneled CVAD such as peripherally inserted central catheter (PICC) or an external tunneled CVAD like, for example, Hickman-Broviac devices. These catheters carry, furthermore, a thrombotic risk, rare in hemophilia but not totally absent. In a meta-analysis of CVAD in hemophilia, pooled incidence of infection was 0.66 per 1000 catheter days, the presence of inhibitors was an independent risk factor for infection and infection was less likely in children >6 years of age and in recipients of fully implanted CVAD. Infection was the reason for CVAD removal in 69.9% of cases and thrombosis in 4.1% [61]. In order to help those giving treatment to make the best choice for their patients, an international consensus conference among hemophilia experts was held in 2004 [62], and the participants stated that, wherever possible, peripheral veins remain the route of choice, and the use of central venous access devices should be limited to cases of clear need in patients with caregivers able to exercise diligence in CVAD care and should continue no longer than necessary.

On the other hand, the good outcome obtained with AVF [60] leads to diversely considering this possibility, but the AVF creation has an anatomical age limit based on the size of the brachial artery and the identification of a suitable vascular site by the vascular surgeon, who should be an expert in operating on small caliber vessels [60].

## 8. Dental Care

Hemophilic children should be educated to a correct oral hygiene, targeted by brushing twice daily with a medium texture bristles toothbrush and age-adapted fluoride toothpaste, and using interdental cleaning aids, such as floss, tape, and interdental brushes to prevent the formation of dental caries and periodontal disease. Where water does not have a fluoride content of at least 1 ppm, fluoride toothpaste as well as additional fluoride supplements should be recommended [6].

Fixed and removable orthodontic appliances may be used along with regular preventive advice and hygiene therapy. However, special care should be taken to ensure that the gengiva is not damaged when fitting the appliance [6].

A number of dental procedures do not require augmentation of coagulation factor levels including examinations, fissure sealants, small occlusal restorations without the need for local anesthesia and supragengival scaling. Treatment can be safely carried out under local anaesthesia using the full range of techniques available to dental surgeons [63]. 

For procedures that do require increment in the factor levels, there may be four therapeutic management options depending on the type of hemophilia, namely:

1. Coagulation factor replacement therapy
2. Release of endogenous factor stores using desmopressin (DDAVP)
3. Improving clot stability by antifibrinolytic drugs, for example tranexamic acid, to reduce the need for replacement therapy
4. Local haemostatic measures (such as suturing, and local measures, such as the use of oxidized cellulose).

For example, infiltration, intra-papillary, and intra-ligamentary injections are often done under factor cover (20–40%), though it may be possible for those with adequate experience to administer these injections without it [63,64]. It is advisable that complicated dental procedures, such as dental extraction or surgical procedures carried out within the oral cavity, should be performed in a Hemophilia Treatment Center. 

## 9. Joint Evaluation

In the frame of a comprehensive care approach, growing attention has been given to the periodic assessment of the joint status in hemophilia patients, with the aim to identify early joint damage and to prevent the development of a clinically overt arthropathy. Besides clinical examination, X-ray and magnetic resonance imaging (MRI) are currently used to evaluate joint status and to monitor the disease progression in hemophilia. In addition, ultrasound examination (US) was demonstrated capable of early detection and monitoring of synovial hypertrophy and osteochondral changes in hemophilia, thus helping to identify joints that need to be studied with a second-level examination such as MRI. On this wave, US was proposed as part of the routine clinical examination by hemophilia experts to optimize the diagnostic workup [65,66,67]. 

## 10. From Day-Care to School

Finding an out-of-home child care center may seem doubly challenging for a parent of a child with a bleeding disorder, but day care or other activity groups can provide stimulation that the child needs, avoiding the risk of overprotection.

The school staff and students should be informed that a child has hemophilia, preferably by the parents. Education of school personnel regarding suitable activities for the child and immediate care in case of bleed is recommended.

If adequate prophylaxis is given, no additional resources are required for medical reasons.

## 11. Sport and Hemophilia in Childhood

Physical exercise and sport is one of the basic foundations in the treatment of hemophilia. Specifically, a child with hemophilia would benefit from exercise and sport, both because a good muscle tone can decrease the frequency of bleeds, joint problems and loss of bone mineral density, and because it can contribute to improving their quality of life [68,69,70,71,72,73]. Furthermore, acute exercise sessions increment the levels of Factor VIII, subsequently improving coagulation in mild patients [73].

Physical activity should be encouraged, with attention paid to muscle strengthening, coordination, general fitness, physical functioning, healthy body weight and self-esteem. The choice of activities should reflect an individual’s preference/interest, ability, physical condition and resources. If non-contact sports (such as swimming, cycling, and walking) should always be encouraged, high contact sport (soccer, rugby, boxing) or high velocity activities (motorcross) are best avoided unless the individual is on good prophylaxis to cover such activities [71]. Patients with moderate or mild hemophilia may experience more bleeding with physical activity since they do not receive prophylaxis [74].

Efforts should be made to maximize safety for patients with hemophilia. 

As a general principle, participation in organized sport programs with adult supervision is better than the practice of unstructured activities. Moreover, the appropriate use of safety equipment should be favored (in some cases, the protection of joints with braces or splints is recommended), proper footwear and the timing of prophylaxis should be carefully personalized (to maximize the factor level at the time of sport participation) [6,74].

## 12. Adolescence

Persons with hemophilia, living with their condition from infancy, require attention from a biopsychosocial approach. They may benefit greatly from having professional help to achieve the best quality of life possible setting up tailored objectives throughout the patient’s life, including disease control, addressing the particular difficulties, and achieving optimal empowerment. This becomes even more critical in prepuberty and adolescence, as these periods are considered at risk from a clinical-biological point of view (such as overweight) and a psychological point of view (such as psychosexual and psychosocial regression) [75]. The adherence to self-administered therapy in adolescent patients is not always complete; on the other hand, overprotection is to be avoided [72,75]. In addition, the transition of care to the adult center may represent a critical phase, and should include at least one joint meeting with the pediatric and adult team [75]. 

## 13. Psychological Issues

Hemophilia does not predispose to any mental illness, but the person with hemophilia and his environment may greatly benefit from having professionals help them manage to adapt to the disease, cope with the experience of suffering and overcome the difficulties caused by chronicity, achieving the best quality of life (QoL) possible [76]. Psychosocial and cultural factors exert an influence on patients’ QoL and the cultural background plays an important role [77]. Psychosocial factors affecting QoL include coping, social support and locus of control that may influence both as resources and stress factors [78].

When a child is initially diagnosed, shock, denial, anger and depression are common emotional responses of the parents [79] that play a significant role in the care of a child with hemophilia and refers to experiencing a large responsibility for management of hemophilia at home [80]. Parents have to face not only feelings of anxiety, guilt and worry over their child’s condition, but also the impact of pain during infusion [81].

Between fathers and mothers, the last ones are usually more involved in the daily care for their child and this condition could predispose to psychosocial problems [82]. Moreover, if there are unaffected siblings, the risk for them is to not get enough of their parents’ time, which could neglect their needs [83], and they may need help to overcome the “healthy sibling syndrome” where the healthy child feels guilty that he/she is healthy. 

The model of family reorganization after diagnosis is essential for the child to develop his own cognitive model of adjustment to the disease. Communication with the healthcare team could promote, at this stage, new strategies of problem solving in parents, enhancing their self efficacy and empowerment [76]. For example, at the beginning, it is usual that the child is overprotected, but this behavior is not useful for the child’s psychological and social development and the team could help parents to adapt their strategies. 

Children with hemophilia do not all have the same psychological and emotional experiences. As the child grows up, he should be encouraged to talk about hemophilia to promote an adaptive cognitive construction of the disease and its management. A critical point is the prophylaxis: a greater adherence is achieved during infancy and childhood [84], even though some challenges exist: one of them is represented by the development of inhibitors. During adolescence, there are some important changes (physical, psychological and social) that could affect the previous adjustment. At this stage, complications and severe physical sequelae may occur as a result of disease complication or neglect of bleeding symptoms [75] because of psychological mechanisms such as denial.

In conclusion, it is important to highlight that the way to react to illness is unique and the specialist should follow a multidimensional perspective, understanding the significance of the disease situation in each family [85].

## 14. Conclusions

Hemophilia care in the pediatric age is a multidisciplinary task. It requires the contribution of the hematologist, specialized in hemostasis and thrombosis with experience in pediatric patients, the surgeon with experience of CVADs in children, the psychologist and social worker, the pharmacist, the orthopedic, physiatrist and physiotherapist, and the nurse team to assist the patient and his family on a regular basis. It is of great importance to establish a liaison with the family and the child with hemophilia, in order to promote trust, reliability and good communication between the family and their caregivers. Setting this basis in the pediatric age can have an impact on the disease outcome in adult age. The goal must be to avoid bleeding complications and joint damage in the pediatric age in order to enable the hemophiliac patient to reach adulthood as healthy as possible. 

## Figures and Tables

**Table 1:** Definition of factor replacement protocol and prophylaxis regimen choice [6].

Protocol		Prophylaxis Regimen Choice
Continuous prophylaxis		If initiated with the intent of treating for 52 weeks of the year and is accomplished for at least 45 weeks of that year
	Primary prophylaxis	The regular, continuous use of treatment that is started before the second clinically evident large joint bleed (bleeds in ankles, knees, hips, elbows, or shoulders). It is initiated before three years of age in children without documented osteochondral joint disease (determined by a physical examination and/or imaging studies)
	Secondary prophylaxis	Initiated after ≥2 bleeds into large joints but before the onset of joint disease documented by physical examination and imaging studies
	Tertiary prophylaxis	The continuous treatment started after the onset of joint disease, as documented by physical examination and plain radiographs of the affected joints
Intermittent prophylaxis		If the prophylaxis regimen does not exceed more than 45 weeks in a year
“On-demand” therapy		Treatment given at the time of clinically evident bleeding
